# Supplementary material for: Efficacious human metapneumovirus vaccine based on AI-guided engineering of a closed prefusion trimer
Source: Nat Commun. 2024 Jul 25;15:6270. doi: 10.1038/s41467-024-50659-5 (PMC11272930; doi:10.1038/s41467-024-50659-5)
Supplement: Supplementary file 1 — Supplementary Information [file 41467_2024_50659_MOESM1_ESM.pdf]

Supplemental data to:

Efficacious human metapneumovirus vaccine based on AI-guided engineering of a closed prefusion trimer

Authors

Mark J.G Bakkers[1,2], Tina Ritschel[1,3], Machteld Tiemessen[1], Jacobus Dijkman[1,4,5], Angelo A. Zuffianò[1,6], Xiaodi Yu[7], Daan van Overveld[1], Lam Le[1], Richard Voorzaat[1], Marlies van Haaren[1], Martijn de Man[1], Sem Tamara[1], Leslie van der Fits[1], Roland Zahn[1], Jarek Juraszek[1,$], Johannes P.M Langedijk[1,2,$,#]

Affiliations:

[1] Janssen Vaccines & Prevention BV, Leiden, The Netherlands
[2] Current affiliation: ForgeBio B.V. Amsterdam, The Netherlands
[3] Current affiliation J&J Innovative Medicine Technology, R&D
[4] Current affiliation: Van 't Hoff Institute for Molecular Sciences, University of Amsterdam, The Netherlands
[5] Current affiliation: AMLab, AI4Science Lab Informatics Institute, University of Amsterdam, The Netherlands
[6] Current affiliation: Promaton BV, Amsterdam, The Netherlands
[7] Structural & Protein Science, Janssen Research and Development, Spring House, PA 19044, USA

$ These authors contributed equally to the work
#Correspondence to: hlangedijk@forge-bio.com

This file includes:

Figures S1 to S11
Table S1

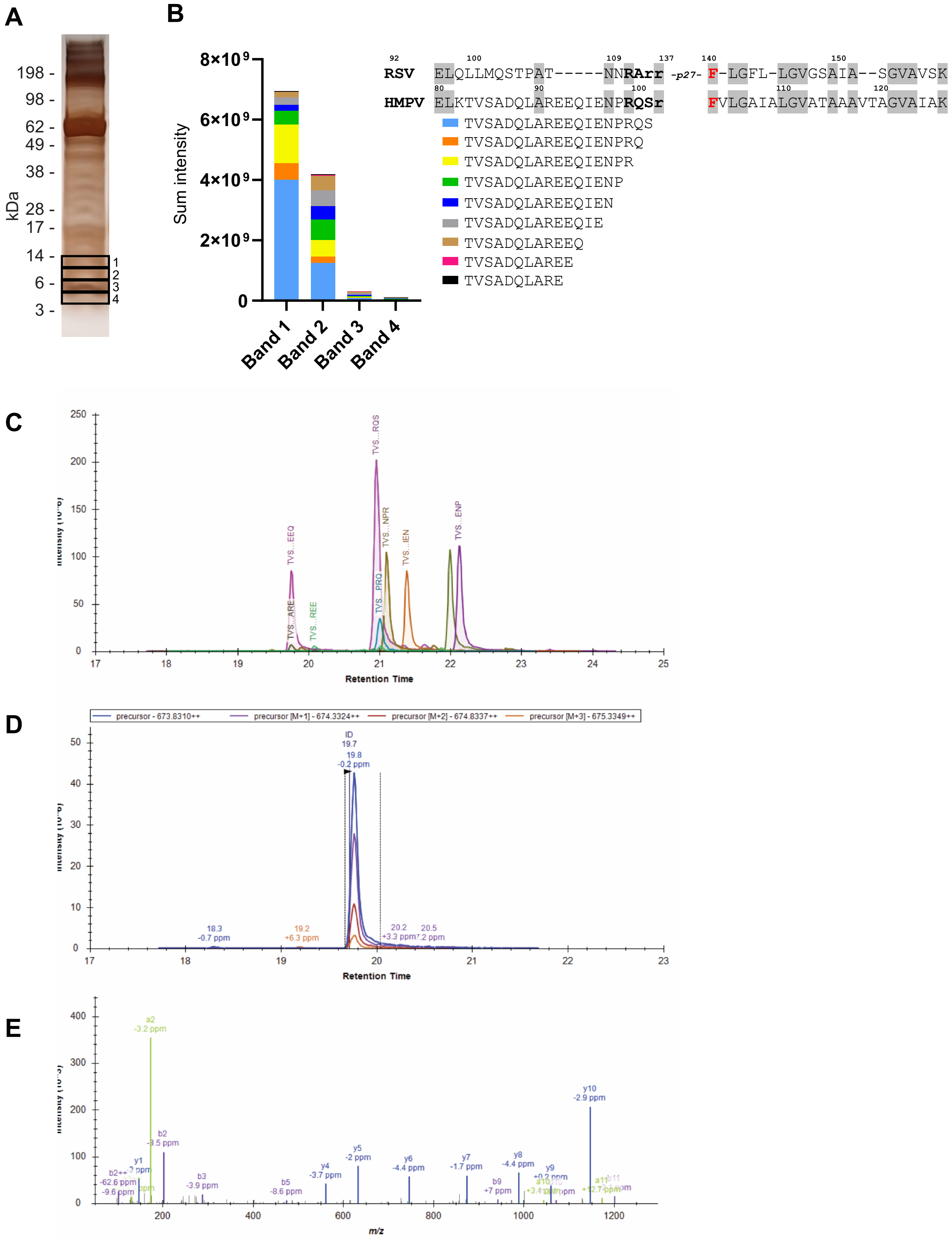

Supplementary Figure 1. LC-MS identification of C-terminal peptides of HMPV F2 protein. (A) Silver stained reduced SDS-PAGE of supernatants from HMPV infected hAECs, 4 days after infection. Black rectangles indicate the excised bands that were subjected to in gel digestion with Lys-C. (B). Peptides eluted from excised bands were analyzed by liquid chromatography-mass spectrometry (LC-MS/MS) to determine the identity of the C-terminal F2 peptide variants. The absolute abundances of all detected C-terminal F2 peptide variants were summed and the percentage of each peptide variant was calculated. (C) Extracted ion chromatograms of HMPV F2 C-terminal peptides in band 2. (D) Extracted ion chromatogram of doubly-charged TVSADQLAREEQ peptide plotted for the four most abundant isotopic peaks. Integration window is indicated with vertical dashed lines. Peaks are annotated with apex retention time in minutes and precursor mass difference from theoretical in ppm. (E) Annotated fragmentation spectrum for TVSADQLAREEQ peptide with mass error of each fragment and fragment name displayed above the peaks.

A

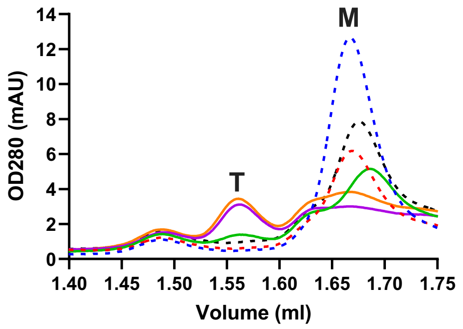

	cleaved	truncation	HRB stabilization
1	-	-	-
2	yes	yes	-
3	yes	yes	(II) L473W, S477I
4	yes	yes	(III) L473W, S477I, A484I
5	yes	yes	(VII) L473W, D475R, Q476K, S477F, N478D, R479E, A484I
6	yes	-	(VII) L473W, D475R, Q476K, S477F, N478D, R479E, A484I

B

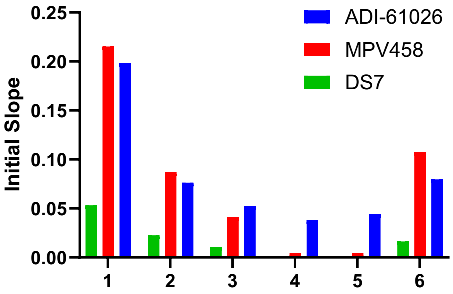

	cleaved	truncation	HRB stabilization
1	-	-	-
2	yes	yes	-
3	yes	yes	(II) L473W, S477I
4	yes	yes	(III) L473W, S477I, A484I
5	yes	yes	(VII) L473W, D475R, Q476K, S477F, N478D, R479E, A484I
6	yes	-	(VII) L473W, D475R, Q476K, S477F, N478D, R479E, A484I

C

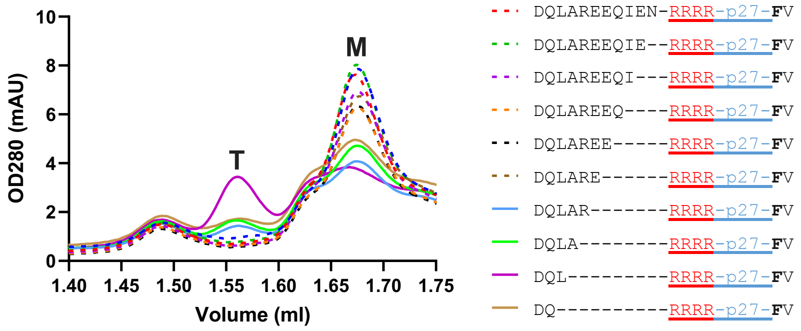

D

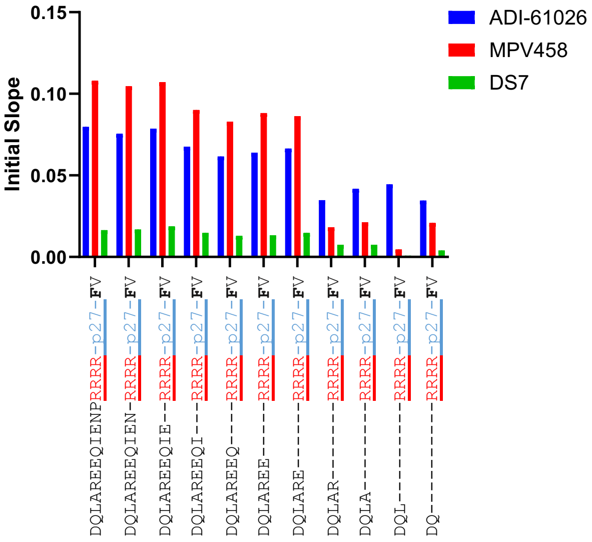

Supplementary Figure 2. Supernatant screen of HMPV F protein to identify the effect of truncation and HR2 stabilization; raw data of Figure 3. (A) Analytical SEC on HMPV F variants in supernatant that differ in HR2 stabilization. The trimer (T) and monomer (M) peaks are indicated. The table on the right describes the different stabilizations applied. (B) Expression of HMPV F variants from (A) in supernatant as measured by BLI using immobilized antibodies ADI-61026 (blue), MPV458 (red) and DS7 (green). The initial slope V0 at the start of binding is shown. (C) Analytical SEC on HMPV F variants in supernatant that differ in F2 truncation. The table on the right describes the F2 truncations. (D) Expression of HMPV F variants from (C) in supernatant as measured by BLI using immobilized antibodies ADI-61026 (blue), MPV458 (red) and DS7 (green).

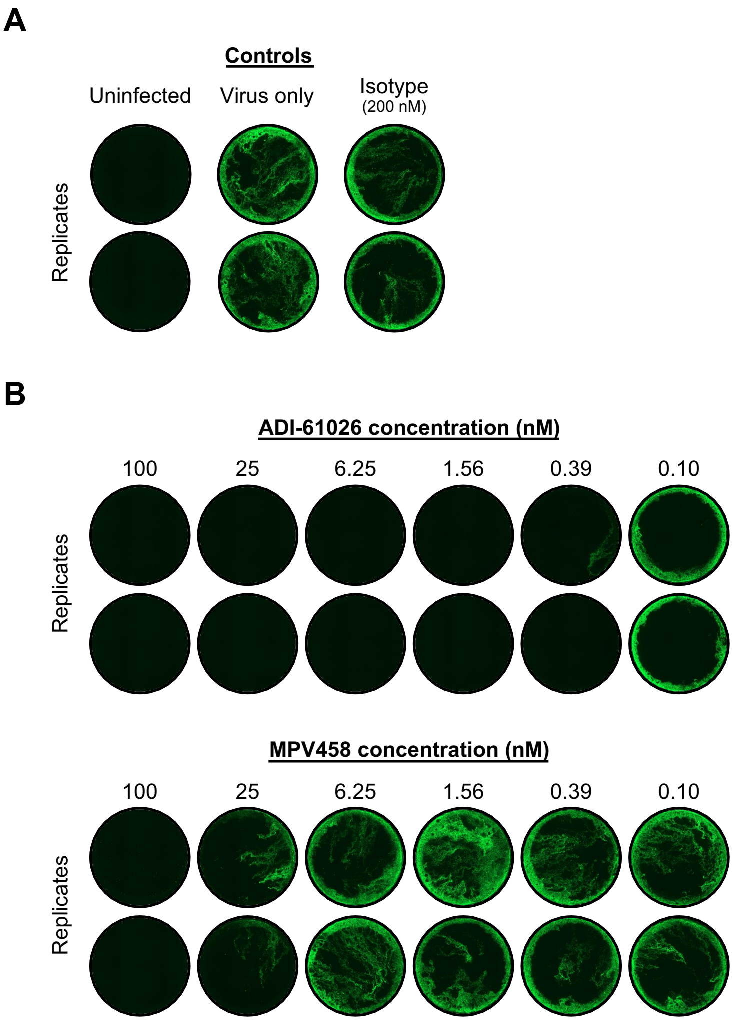

Supplementary Figure 3. Virus neutralization assay (VNA) using differentiated primary human airway epithelial cells (hAEC). (A) Control hAEC inserts were either uninfected, or infected in the absence ('virus only') or presence ('isotype') of IgG isotype control at 200 nM, using the hMPV CAN97-83-GFP virus strain. The GFP signal across the full insert is shown. (B) Dose-dependent neutralizing activity of ADI-61026 and MPV458 against hMPV CAN97-83-GFP strain. Data is visualized as in (A).

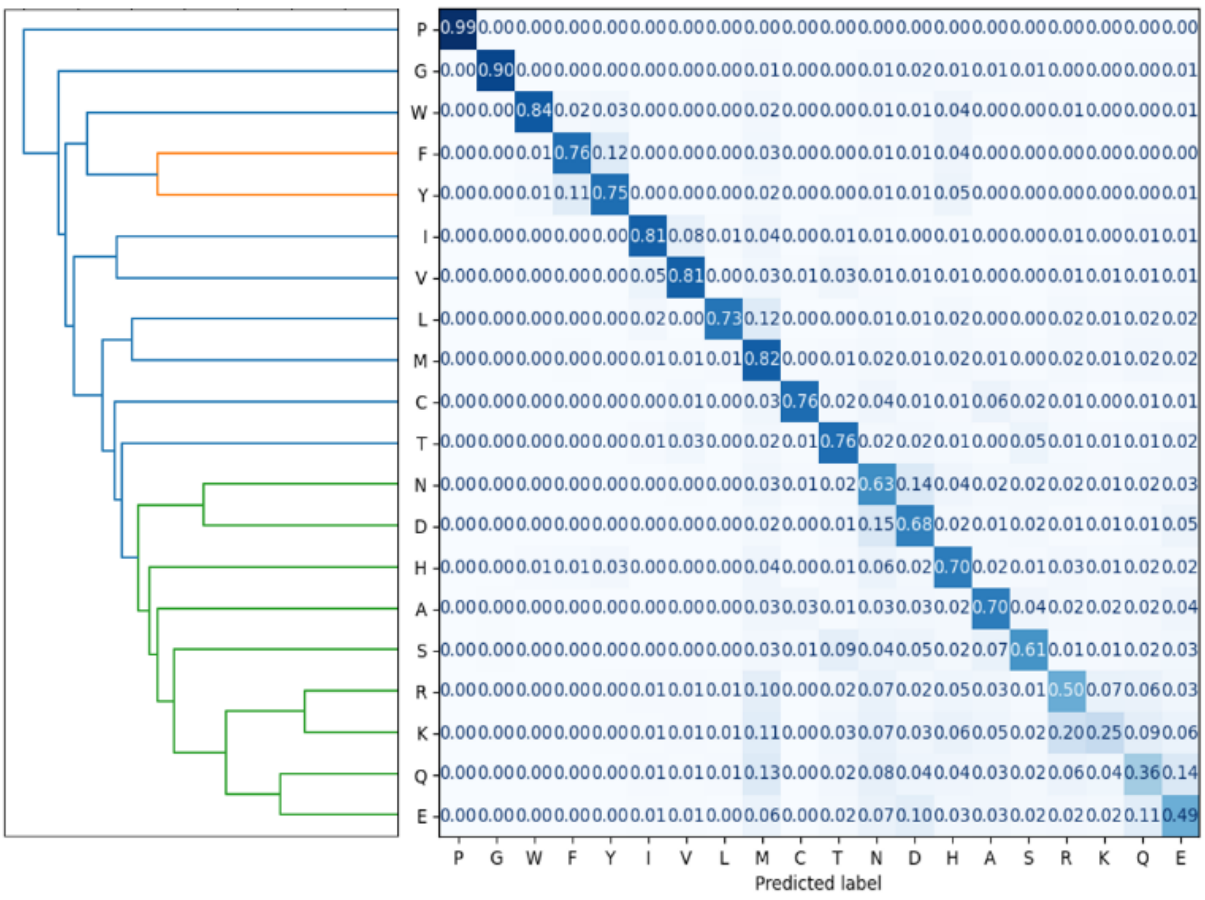

Supplementary Figure 4. Hierarchical cluster of the normalized confusion matrix.

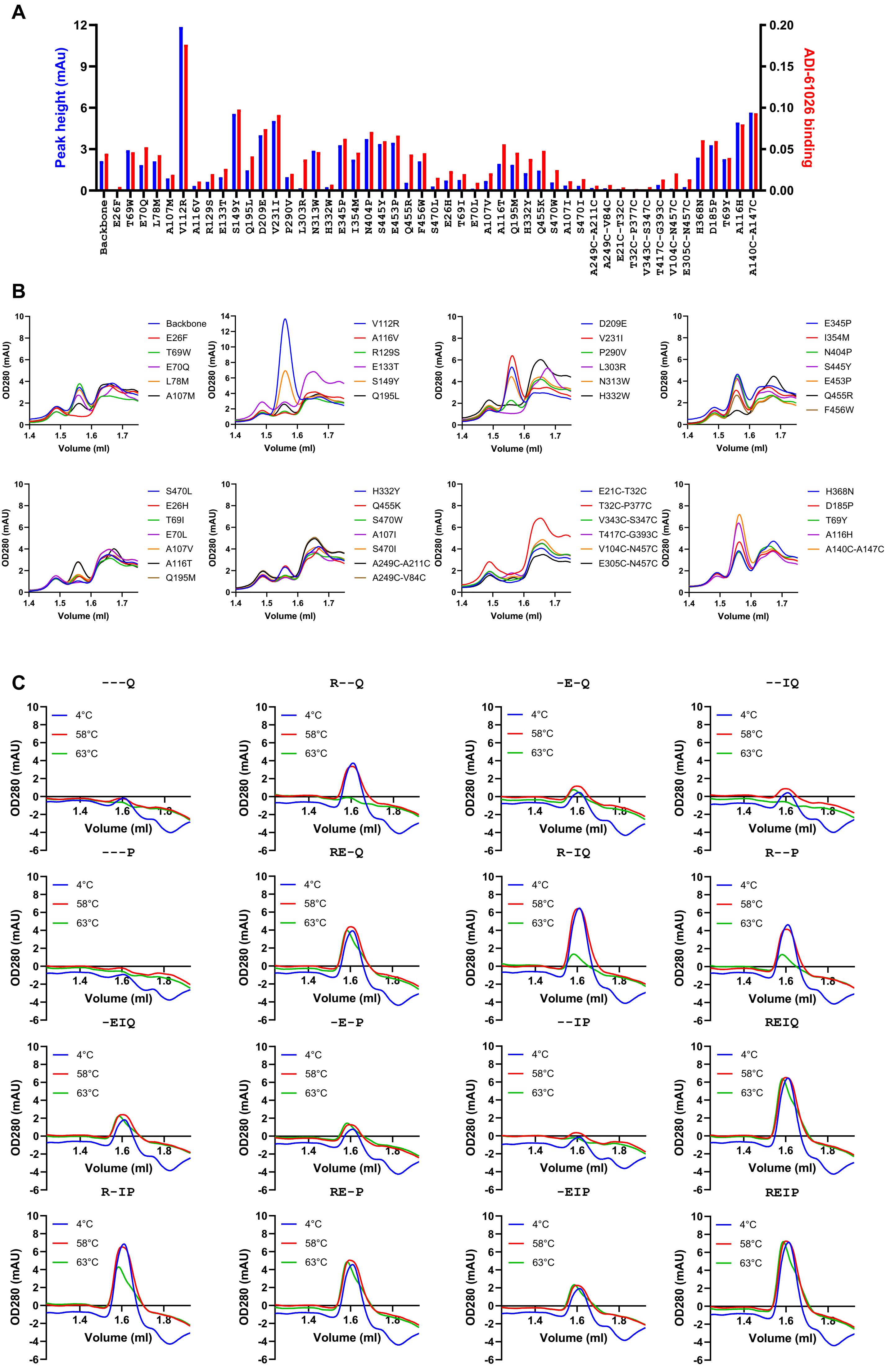

Supplementary Figure 5. Identification of HMPV Pre-F stabilizing substitutions. (A) The peak height of HMPV F2 trimers derived from analytical SEC (blue bars) together with the expression of these same designs in supernatant as measured by BLI using immobilized ADI-61026 (red bars). The initial slope V0 at the start of binding is shown. (B) Analytical SEC on HMPV F variants in supernatant incorporating point mutations. (C) Analytical Heat-SEC on HMPV F designs incorporating combinations of stabilizing mutations. Supernatants were analyzed by analytical SEC after incubation at 4, 58 or 63°C for 15 min.

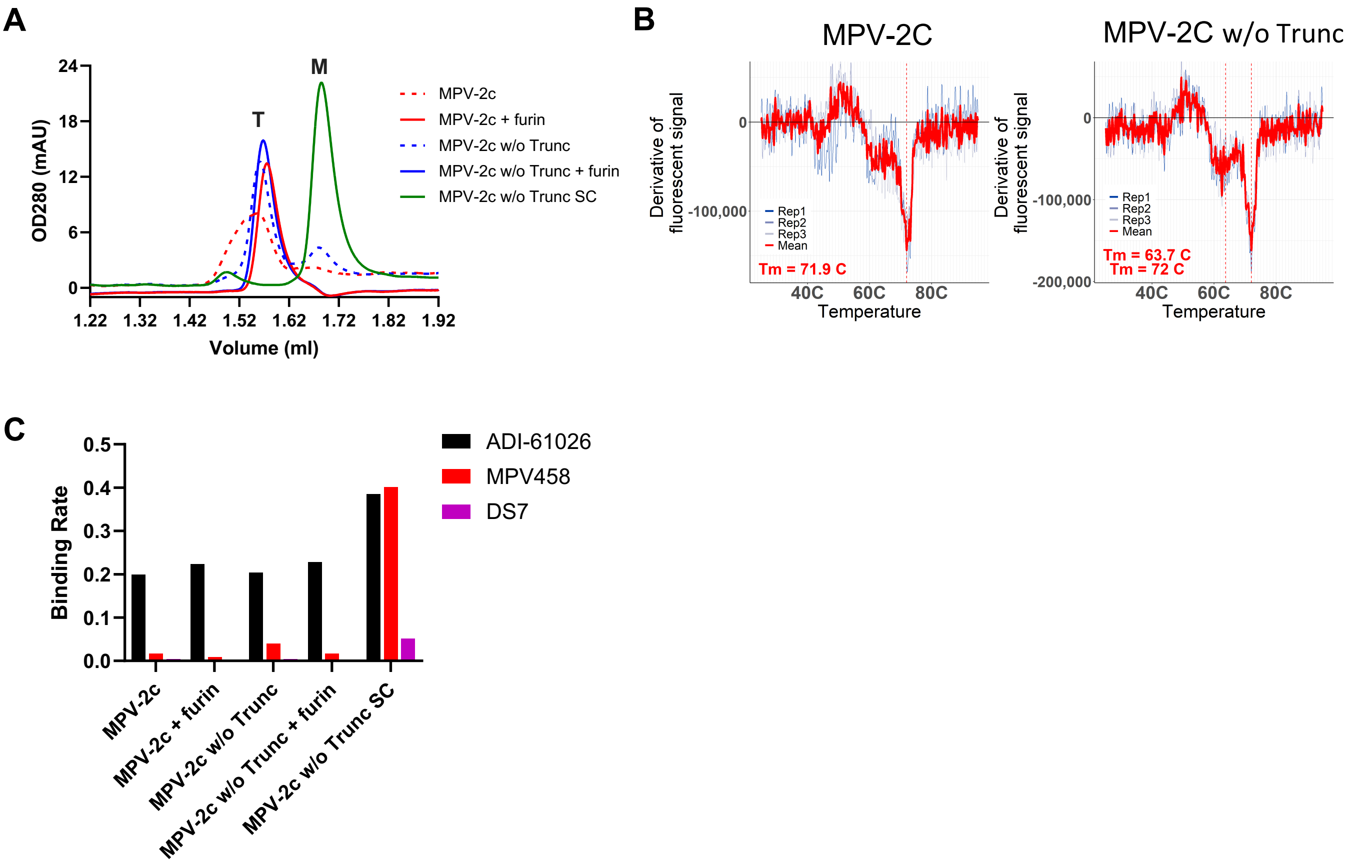

Supplementary Figure 6. Analysis of non-cleaved, single-cleaved and double-cleaved stabilized hMPV Pre-F variants (A) Analytical SEC on HMPV F variants (HR2-VII), stabilizing substitutions V112R, D209E, V231I and E453P which is either uncleaved, cleaved or double cleaved in supernatant with or without (w/o) 20% furin co-expression. (B) Temperature stability of the hMPV PreF variants using DSF. (C) Conformation of the proteins as assessed by BLI using mAb DS7 and prefusion-specific mAbs MPV458 and ADI-61026.

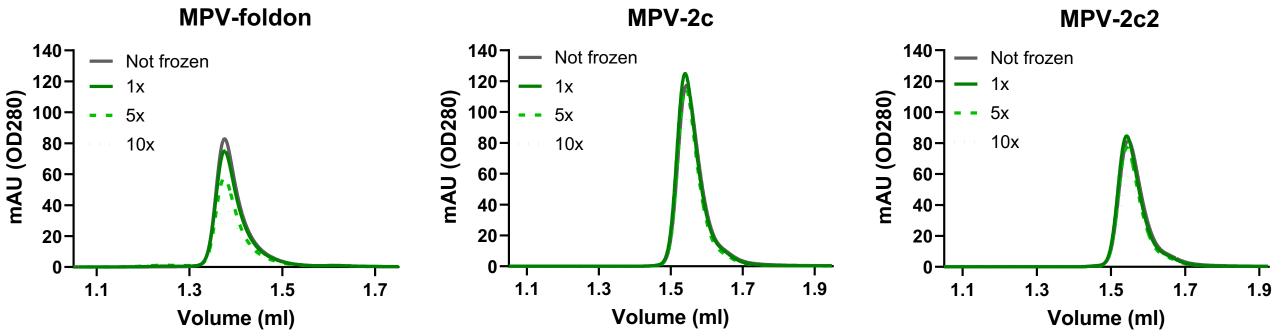

Supplementary Figure 7. Freeze-thaw stability of purified HMPV Pre-F proteins. Analytical SEC traces of purified HMPV F2 proteins after being subjected to indicated number of freeze-thaw cycles. An unfrozen sample ('not frozen'), is taken along as positive control.

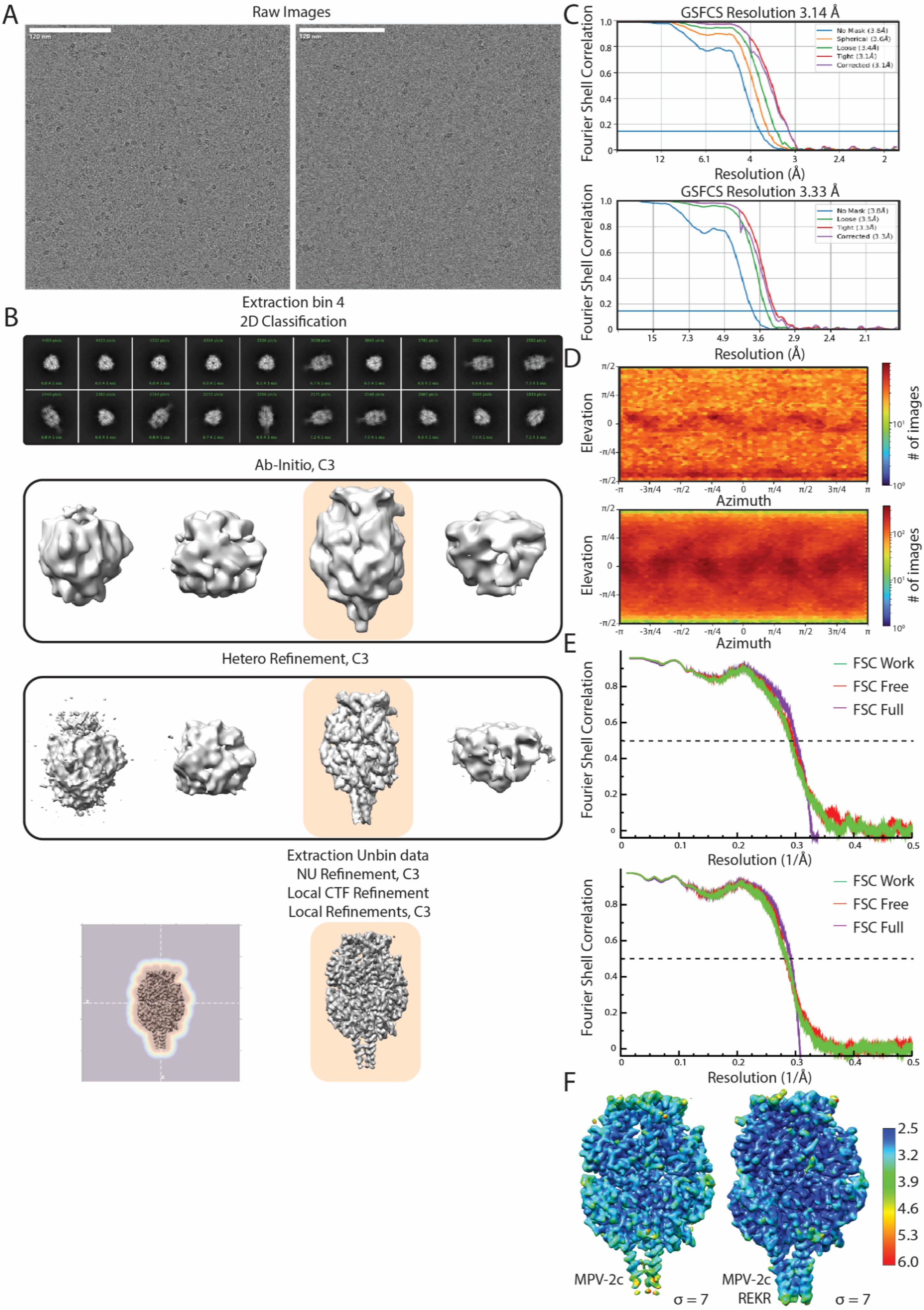

Supplementary Figure 8. Cryo-EM analysis of HMPV prefusion ternary complex. (A) Reference raw images. (B) Flow chart of the cryo-EM data processing procedure. Details can be found in the Materials and Methods. (C) Fourier shell correlation (FSC) curves of the HMPV prefusion ternary complex structures at neutral or low pH with FSC as a function of resolution using CryoSPARC Local refinement outputs, respectively. (D) Angular orientation distribution of the particles at neutral or low pH used in the final reconstructions. The particle distribution is indicated by different color shades. (E) Comparison of the FSC curves between model and half map 1 (work), model and half map 2 (free), and model and full map are plotted in green, red, and magenta, respectively. (F) Local resolution of the maps and colored as indicated.

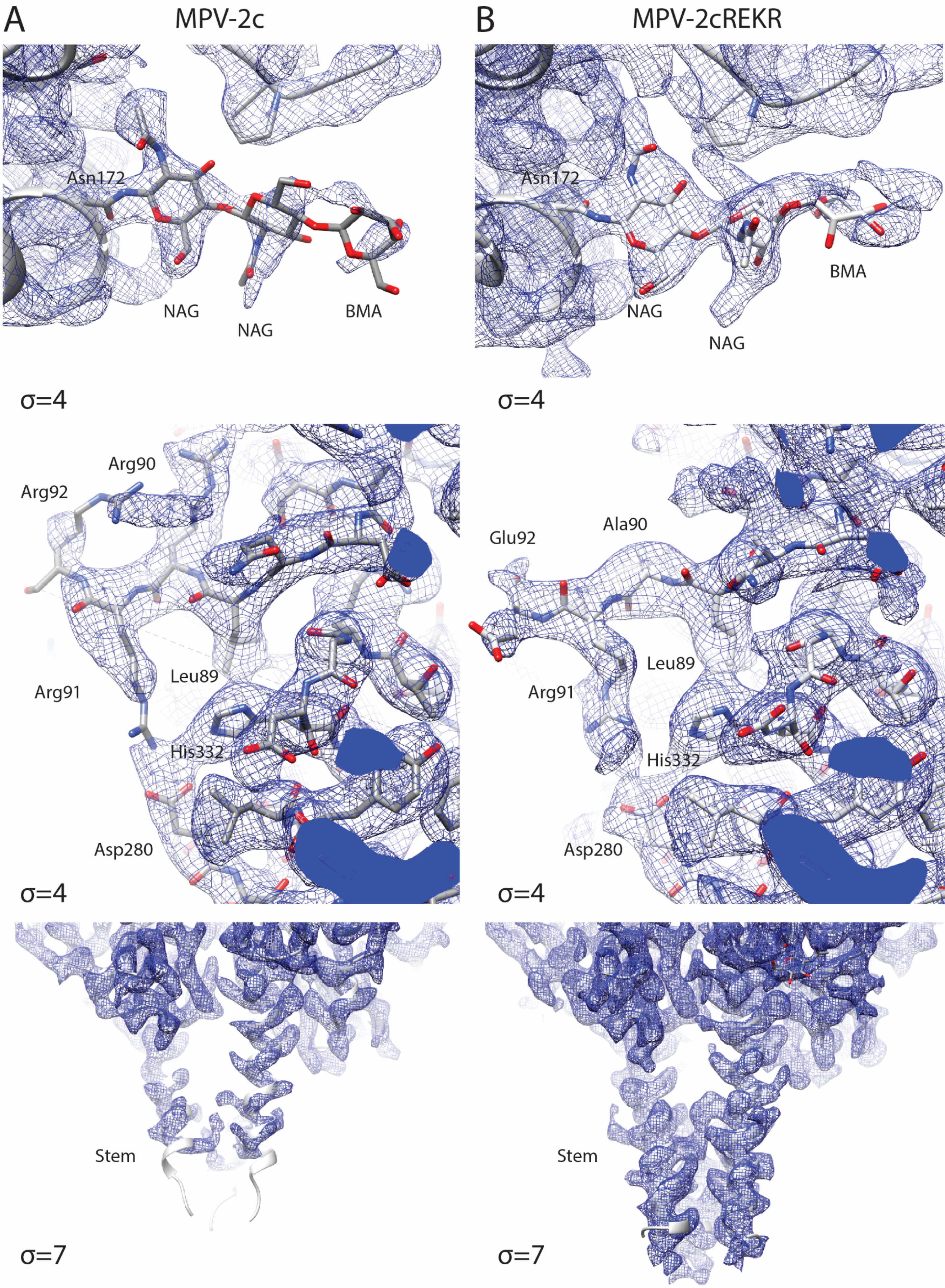

Supplementary Figure 9, Local Cryo-EM densities at Asn172, cleavage site, and stem regions from MPV-2c (A) and MPV-2cREKR (B) structures, respectively.

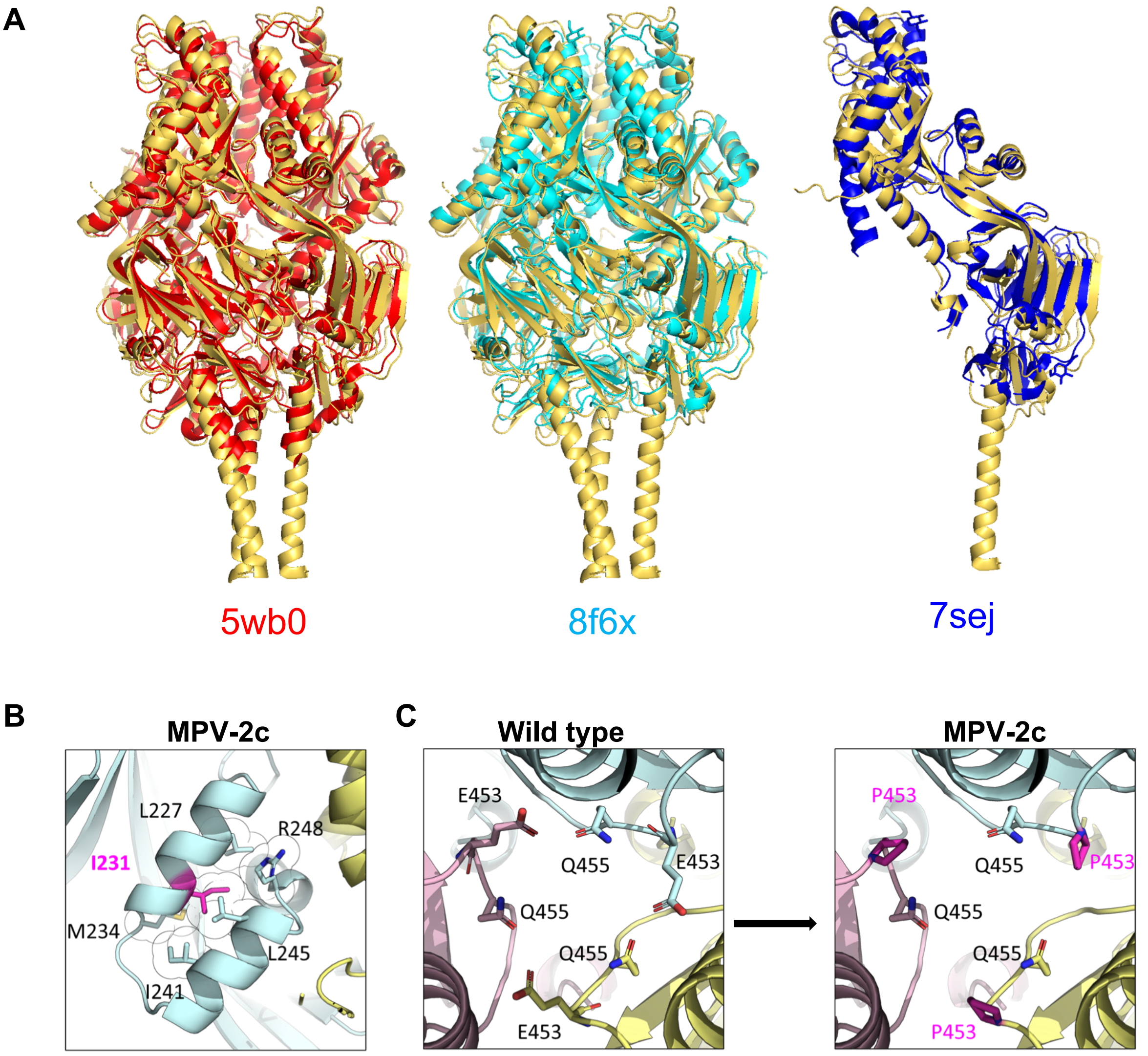

Supplementary Figure 10 (A) structural comparison of MPV-2c (golden) with stabilized cleaved (5wb0) or single chain (8f6x) PreF trimers and a stabilized Pre-F trimer solved as a monomer (7sej). All trimers except for MPV-2c are C-terminally fused to a heterologous foldon trimerization domain. (B) Sidechain packing of I231 with amino acid surfaces indicated with black lines to illustrate sidechain packing. (C) Top view down the trimeric symmetry axis of region around position 453 in wild-type (left) and the stabilizing substitution E453P in the turn in HR2 (right). Stabilizing substitutions are shown in magenta

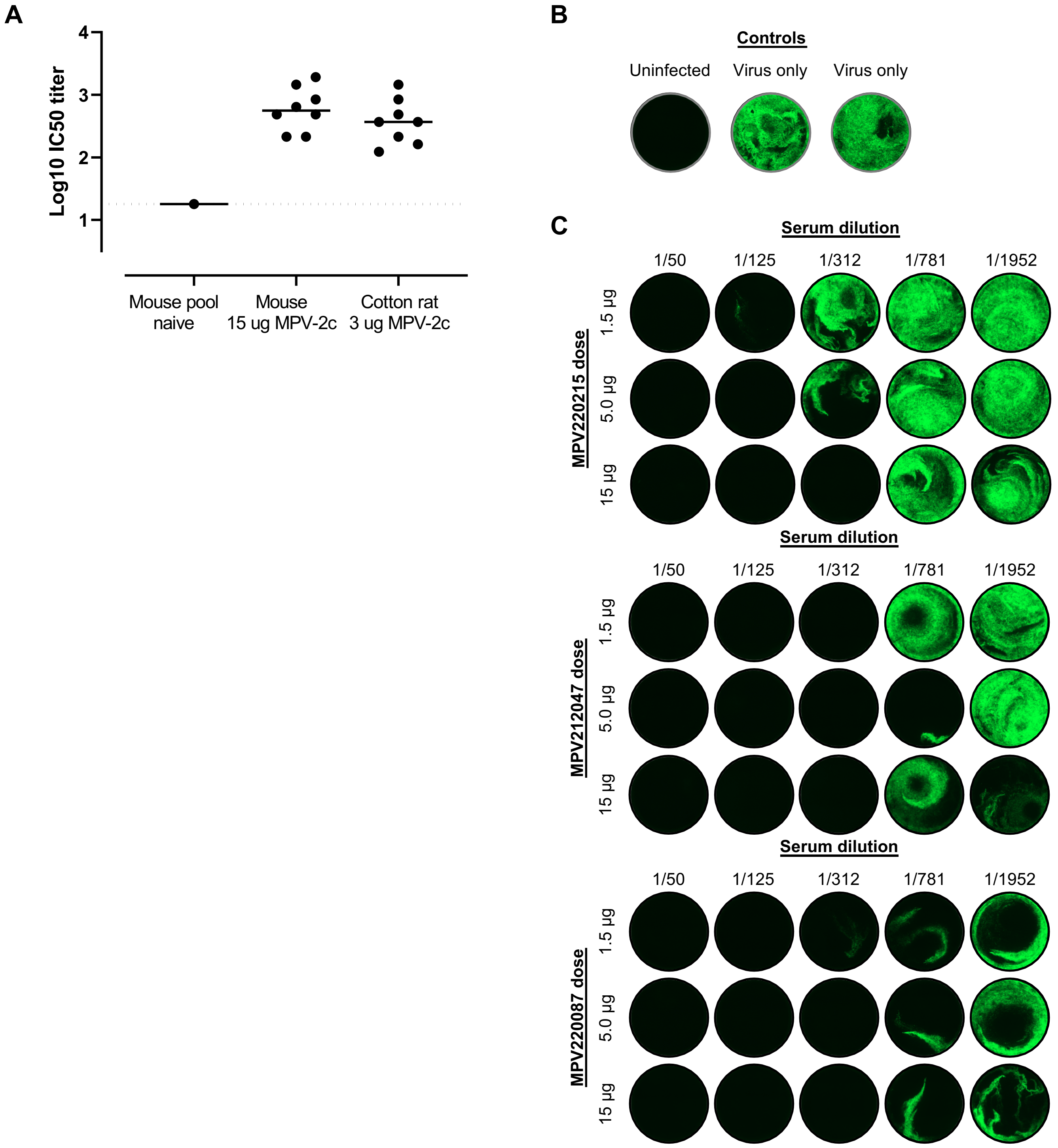

Supplementary Figure 11. Additional VNA analysis on preclinical samples. (A) VNA using hMPV B1 strain to detect cross neutralizing antibodies in immunized mouse and cotton rat sera. (B, C) Virus neutralization assay (VNA) using differentiated primary human airway epithelial cell (hAEC) cultures. (B) Control hAEC inserts were either uninfected or infected in the absence (‘virus only’) of serum, using the hMPV CAN97-83-GFP virus strain. The GFP signal across the full insert is shown. (C) Dose-dependent neutralizing activity of indicated sera against hMPV CAN97-83-GFP strain. Data is visualized as in (B).

Supplementary Table 1. Data collection, reconstruction, and model refinement statistics

	MPV-2c<br><br>EMD-43516, PDB: 8VT2	MPV-2cREKR<br><br>EMD-43517, PDB: 8VT3
**Data collection**		
Microscope	Glacios	Glacios
Voltage (keV)	200	200
Nominal magnification	150,000 x	150,000 x
Exposure navigation	Image Shift	Image Shift
Electron exposure (e /Å^2)	40.0	40.0
Dose rate (e/pixel/sec)	5.3	5.3
Detector	Falcon III	Falcon IV
Pixel size (Å)*	0.948	0.91
Defocus range (µm)	-0.8 to -2.4	-0.8 to -2.4
Micrographs Used	2,548	6,144
Final Refined particles (no.)	97,386	453,003
**Reconstruction**		
Symmetry imposed	C3 (NU Refinement)	C3 (NU Refinement)
Resolution (global)		
FSC 0.143	3.14 Å	3.33 Å
Applied B-factor (Å^2)	-128.2	-127.2
**Refinement**		
R.m.s deviations		
Bond lengths (Å)	0.0135	0.0112
Bond angles (º)	0.91	0.82
Ramachandran		
Outliers	0.00 %	0.00 %
Allowed	7.70 %	1.61 %
Favored	92.30 %	91.87 %
Poor rotamers (%)	0.00 %	0.00 %
MolProbity score	1.85	1.70
EMRinger score	3.85	3.85
Clashscore (all atoms)	6.86	4.26

*Calibrated pixel size at the detector

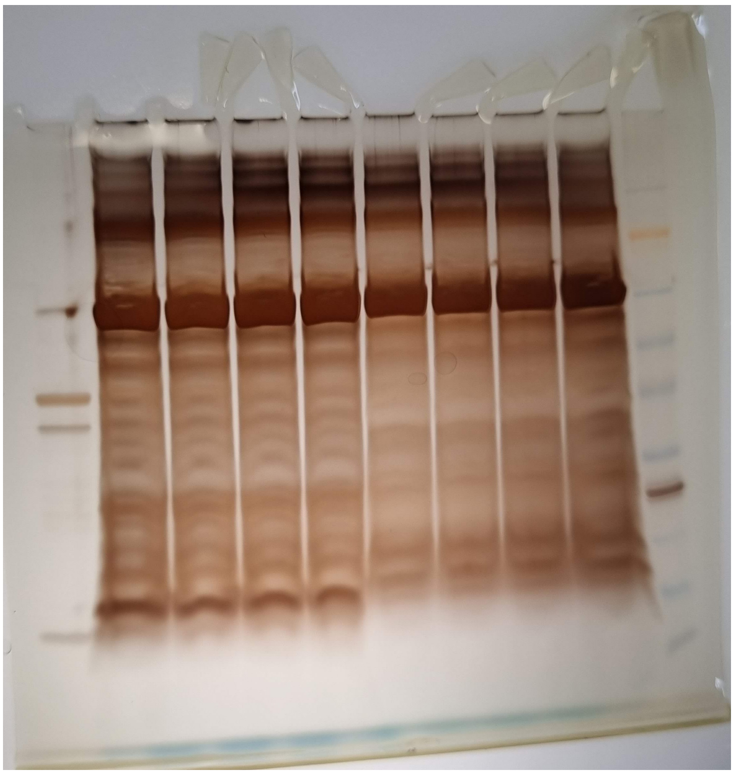

Uncropped blot of sFig 1A
